# A nomogram based on HBeAg, AST, and age to predict non-minimal liver inflammation in CHB patients with ALT <80 U/L

**DOI:** 10.3389/fimmu.2022.1119124

**Published:** 2023-01-19

**Authors:** Lu Zhang, Xiaoyue Bi, Xiaoxue Chen, Luxue Zhang, Qiqiu Xiong, Weihua Cao, Yanjie Lin, Liu Yang, Tingting Jiang, Wen Deng, Shiyu Wang, Shuling Wu, Ruyu Liu, Yuanjiao Gao, Ge Shen, Min Chang, Hongxiao Hao, Mengjiao Xu, Leiping Hu, Yao Lu, Minghui Li, Yao Xie

**Affiliations:** ^1^ Department of Hepatology Division 2, Beijing Ditan Hospital, Capital Medical University, Beijing, China; ^2^ Infectious Disease Department, Xuanwu Hospital, Capital Medical University, Beijing, China; ^3^ Department of General Surgery, Beijing Ditan Hospital, Capital Medical University, Beijing, China; ^4^ Department of Infectious Diseases, Miyun Teaching Hospital, Capital Medical University, Beijing, China; ^5^ Department of Hepatology Division 2, Peking University Ditan Teaching Hospital, Beijing, China

**Keywords:** HBV, nomogram, aspartate aminotransferase (AST), hepatitis B e antigen (HBeAg), liver inflammation

## Abstract

**Objective:**

Precise assessment of liver inflammation in untreated hepatitis B e antigen (HBeAg)-positive patients with chronic hepatitis B virus (HBV) infection can determine when to initiate antiviral therapy. The aim of this study was to develop and validate a nomogram model for the prediction of non-minimal liver inflammation based on liver pathological injuries combined with age and alanine aminotransferase (ALT), aspartate aminotransferase (AST), hepatitis B surface antigen (HBsAg), HBeAg, and HBV DNA quantification.

**Methods:**

We retrospectively included 735 HBeAg-positive chronic hepatitis B (CHB) patients with ALT < 80 U/L as the primary cohort and prospectively enrolled 196 patients as the validation cohort. Multivariate logistic regression analysis identified independent impact factors. A nomogram to predict significant liver inflammation was developed and validated.

**Results:**

Multivariate logistic regression analysis showed that HBeAg, AST, and age were independent risk factors for predicting non-minimal liver inflammation in untreated CHB patients. The final formula for predicting non-minimal liver inflammation was Logit(*P*) = −1.99 − 0.68 × Log_10_HBeAg + 0.04 × Age + 0.06 × AST. A nomogram for the prediction of non-minimal liver inflammation was established based on the results from the multivariate analysis. The predicted probability of the model being consistent with the actual probability was validated by the calibration curves, showing the best agreement in both the primary and validation cohorts. The C-index was 0.767 (95%CI = 0.734–0.802) in the primary cohort and 0.749 (95%CI = 0.681–0.817) in the prospective validation cohort.

**Conclusions:**

The nomogram based on HBeAg, AST, and age might help predict non-minimal liver inflammation in HBeAg-positive CHB patients with ALT < 80 U/L, which is practical and easy to use for clinicians.

## Introduction

Some patients with chronic hepatitis B (CHB) can progress to hepatocellular carcinoma directly without hepatic cirrhosis because of hepatitis B virus (HBV) integration and specific HBV mutants, among others (1–3). The pathogenesis of hepatitis B is very complex, involving changes in immunity and cytokines (4, 5). With the advent of new antiviral drugs, such as tenofovir (TDF), tenofovir alafenamide (TAF), and the combination of interferon with nucleo(s)tide analogues for the treatment of CHB, the HBV virological response or the rate of clinical cure has increased significantly (6–9). Currently, it is recommended in many guidelines that CHB patients with a family history of liver cirrhosis and hepatocellular carcinoma should be considered for antiviral treatment after the age of 30–40 years (10–12).

It is essential to evaluate whether liver inflammation has reached the status necessitating antiviral therapy. Alanine aminotransferase (ALT), aspartate aminotransferase (AST), HBV DNA, hepatitis B surface antigen (HBsAg), and hepatitis B e antigen (HBeAg) can be used to predict significant liver inflammation (13–16). After chronic HBV infection, with increasing age, liver inflammation becomes gradually obvious, which means that the immune tolerance has been broken and that it is time to start antiviral therapy. There are different criteria to determine the timing of the breakdown of immune tolerance in patients with CHB infection, two of which are obvious inflammation in the liver biopsy, which is considered the gold standard, and ALT > 2 upper limit of normal (ULN, 80 U/L) lasting 6 months, excluding common diseases such as drugs and fatty liver, among others. Liver biopsy is hindered by poor patient compliance and low reproducibility, which limited its application value for dynamic monitoring during follow-up. Therefore, the use of more serum markers is needed to help determine the severity of liver inflammation.

The main purpose of the present study was to develop a new nomogram for better prediction of non-minimal liver inflammation in CHB patients with ALT < 2 ULN. The study has great importance for clinicians in determining whether treatment-naive patients with CHB infection and with minimal ALT elevation (<80 U/L) need to start antiviral therapy.

## Materials and methods

### Patients

We retrospectively included 735 HBeAg-positive CHB patients in the primary cohort from January 2008 to December 2017. The prospective cohort of 196 patients was enrolled from January 2018 to December 2019 for external validation. Patients underwent liver biopsy at Beijing Ditan Hospital of Capital Medical University. All patients gave written informed consent. This study has been registered in ClinicalTrial.gov (NCT: 04032275).

### Enrollment criteria

The criteria for enrollment were: 1) with CHB infection for more than 6 months; 2) HBeAg positive; 3) not receiving anti-HBV treatment; 4) had liver biopsy; 5) had sustained ALT < 2 ULN; and 6) with HBV DNA ≥10^3^ IU/ml. Patients with HIV infection, other viral hepatitis infections (hepatitis A, C, D, and E), metabolic or drug-associated liver injury, alcoholic liver disease or non-alcoholic fatty liver disease (NAFLD), autoimmune hepatitis, and liver cancer or liver cirrhosis were excluded from the study. Pregnant women were also excluded.

The Knodell scoring system (0–18) of pathology was used to determine liver inflammation from stained specimens by liver biopsy. The enrolled patients were divided into two groups: the minimal liver inflammation (Knodell score, <4 points) group and the non-minimal liver inflammation (Knodell score, ≥4 points) group (17, 18).

### Serological detection

Serum HBsAg and HBeAg were detected using Abbott Architect i2000 test kits (Abbott Park, IL, USA). The detection range of HBsAg was 0.05–250 IU/ml. The sample was diluted to 1:50–1:500 if the HBsAg concentration was more than 250 IU/ml. HBeAg positivity was defined as a detection level >1 sample/cutoff (S/CO). The reference range for ALT or AST was 0–40 U/L.

### Liver biopsy

Liver biopsy was performed using a Max-Core puncture needle (BARD, Peripheral Vascular, Inc., Tempe, AZ, USA). A liver biopsy core about 1.5 cm long with 11–15 portal tracts is appropriate for the evaluation of inflammation. Liver biopsy specimens were read independently by three pathologists, and the average score was used as the final score.

### Statistical analysis

SAS 9.2 and R3.02 software were used for statistical analyses. Logistic regression analysis was performed for the analysis of liver inflammation-related factors, and the nomogram method was used for scoring. The bilateral test was adopted for all analyses. A *p* < 0.05 signifies a statistically significant difference. Bootstraps with 200 resamples were used for validation. The concordance index (C-index) was used to evaluate the accuracy of the nomogram. A calibration index was drawn to evaluate the predicted and observed probabilities.

## Results

### Baseline data of the primary and external validation cohorts

The characteristics of the enrolled patients are listed in **Table 1**. The age (33.22 ± 9.33 *vs*.36.65 ± 8.68 years, *p* < 0.001), platelet (PLT) (202.17 ± 52.57 *vs*. 215.74 ± 60.09 × 10^9^/L, *p* = 0.004), and prothrombin activity (PTA) (86.76 ± 8.97% *vs*. 96.22 ± 10.31%, *p* < 0.001) of the primary cohort were lower than those of the validation cohort.

**Table 1:** Characteristics of the HBeAg-positive chronic hepatitis B (CHB) patients with ALT < 80 U/L in the primary and external validation cohorts.

Characteristics	Total	Primary cohort	Validation cohort	Statistics	*p-*value
	(*n* = 931)	(*n* = 735)	(*n* = 196)		
Sex					
Male, *n* (%)	586 (62.94)	475 (64.63)	111 (56.63)	4.24(*χ* ^2^)	0.040
Female, *n* (%)	345 (37.06)	260 (35.37)	85 (43.37)		
Age (years)	33.95 ± 9.30	33.22 ± 9.33	36.65 ± 8.68	−4.64(*t*)	<0.001
Log_10_HBsAg (IU/ml)	3.57 ± 0.99	3.55 ± 0.97	3.63 ± 1.03	−1.04(*t*)	0.299
Log_10_HBeAg (S/CO)	2.63 ± 0.91	2.66 ± 0.88	2.51 ± 1.02	1.96(*t*)	0.051
Log_10_HBV DNA (IU/ml)	6.95 ± 1.46	6.96 ± 1.38	6.93 ± 1.72	0.24(*t*)	0.811
ALT (U/L)	42.4 (29.6–54.6)	42.4 (30.1–54.6)	42.1 (28.0–54.0)	0.48(*Z*)	0.629
AST (U/L)	28.0 (23.0–35.7)	27.9 (22.9–36.1)	29.0 (24.0–34.0)	0.55(*Z*)	0.581
TBIL (μmol/L)	12.6 (9.5–16.4)	12.8 (9.6–16.5)	12.0 (9.5–16.0)	1.25(*Z*)	0.212
ALB (g/L)	46.1 (44.0–48.1)	46.2 (44.1–48.2)	46.0 (43.0–48.0)	0.55(*Z*)	0.579
WBC (10^9^/L)	5.7 (4.8–6.7)	5.7 (4.8–6.7)	5.7 (4.7–6.7)	0.539(*Z*)	0.589
PLT (10^9^/L)	205.03 ± 54.49	202.17 ± 52.57	215.74 ± 60.09	−2.88(*t*)	0.004
PTA (%)	89.13 ± 10.18	86.76 ± 8.97	96.22 ± 10.31	−11.30(*t*)	<0.001

HBsAg, hepatitis B surface antigen; HBeAg, hepatitis B e antigen; S/CO, sample/cutoff; HBV, hepatitis B virus; ALT, alanine aminotransferase; AST, aspartate aminotransferase; TBIL, total bilirubin; ALB, albumin; WBC, white blood cells; PLT, platelet; PTA, prothrombin activity.

### Univariate and multivariate statistical analyses

The results of the univariate analysis showed that nine influencing factors were correlated with non-minimal liver inflammation in the primary cohort (**Table 2**). When age was over 30 and 40 years, non-minimal inflammation was respectively found in 43.24% and 53.07% of patients with CHB infection and ALT < 80 U/L. Age, Log_10_HBsAg, Log_10_HBeAg, Log_10_HBV DNA, ALT, AST, albumin (ALB), and PLT as continuous variables and sex as the binary variable were introduced into the multivariate logistic regression analysis (**Table 3**). The final formula was as follows: Logit(*P*) = −1.99 − 0.68 × Log_10_HBeAg + 0.04 ×Age + 0.06 × AST.

**Table 2:** Univariate logistic regression analysis of the factors of non-minimal liver inflammation in patients from the primary cohort.

	*b*	SE	Wald	*p*-value	OR	95%CI for OR	
						Lower	Upper
Sex	0.34	0.16	4.71	0.030	1.41	1.03	1.92
Age (years)	0.05	0.01	33.32	0.000	1.05	1.03	1.07
Log_10_HBsAg (IU/ml)	−0.21	0.08	6.59	0.010	0.81	0.69	0.95
Log_10_HBeAg (S/CO)	−0.77	0.10	64.46	0.000	0.46	0.38	0.56
Log_10_HBV DNA (IU/ml)	−0.33	0.06	31.76	0.000	0.72	0.64	0.80
ALT (U/L)	0.02	0.00	14.25	0.000	1.02	1.01	1.03
AST (U/L)	0.06	0.01	50.30	0.000	1.06	1.04	1.08
TBIL (μmol/L)	−0.02	0.01	3.69	0.055	0.98	0.95	1.00
ALB (g/L)	−0.08	0.02	14.26	0.000	0.92	0.88	0.96
WBC (×10^9^/L)	0.01	0.01	0.60	0.437	1.01	0.99	1.03
PLT (×10^9^/L)	−0.01	0.00	20.98	0.000	0.99	0.99	1.00
PTA (%)	0.00	0.01	0.07	0.798	1.00	0.98	1.02

HBsAg, hepatitis B surface antigen; HBeAg, hepatitis B e antigen; S/CO, sample/cutoff; HBV, hepatitis B virus; ALT, alanine aminotransferase; AST, aspartate aminotransferase; TBIL, total bilirubin; ALB, albumin; WBC, white blood cells; PLT, platelet; PTA, prothrombin activity. SE, standard error.

**Table 3:** Multivariate logistic regression analysis of the factors of liver inflammation in patients from the primary cohort.

	*b*	SE	Wald	*p*-value	OR	95%CI for OR	
						Lower	Upper
Intercept	−1.99	0.54	13.69	0.000			
Age (years)	0.04	0.01	17.55	0.000	1.04	1.02	1.06
Log_10_HBeAg (S/CO)	−0.68	0.10	46.70	0.000	0.51	0.42	0.62
AST (U/L)	0.06	0.01	53.80	0.000	1.07	1.05	1.08

HBeAg, hepatitis B e antigen; S/CO, sample/cutoff; AST, aspartate aminotransferase. SE, standard error.

### Establishing a simple scoring system for non-minimal liver inflammation

The scoring system for non-minimal liver inflammation was based on the logistic regression analysis. Each variable was converted into a corresponding score. The total score was the sum of the scores from three independent influencing factors. The total score corresponded to a probability of non-minimal liver inflammation. Details of the point assignment for liver inflammation are shown in **Table 4**.

**Table 4:** Point assignment for the nomogram and the prognostic probability of non-minimal liver inflammation in HBeAg-positive chronic hepatitis B (CHB) patients with ALT < 80 U/L.

Age (years)	Points	AST (U/L)	Points	Log_10_HBeAg (S/CO)	Points	Total points	Risk
10	0	5	0	0	6	3	0.1
15	0	10	1	0.5	5	5	0.2
20	1	15	1	1	4	6	0.3
25	1	20	2	1.5	3	8	0.4
30	2	25	3	2	2	8	0.5
35	2	30	4	2.5	2	9	0.6
40	3	35	4	3	1	10	0.7
45	3	40	5	3.5	0	12	0.8
50	4	45	6			14	0.9
55	4	50	6				
60	5	55	7				
65	5	60	8				
		65	9				
		70	9				
		75	10				

HBeAg, hepatitis B e antigen; S/CO, sample/cutoff; HBV, hepatitis B virus; ALT, alanine aminotransferase.

### Establishing a prognostic nomogram for non-minimal liver inflammation

**Figure 1:**
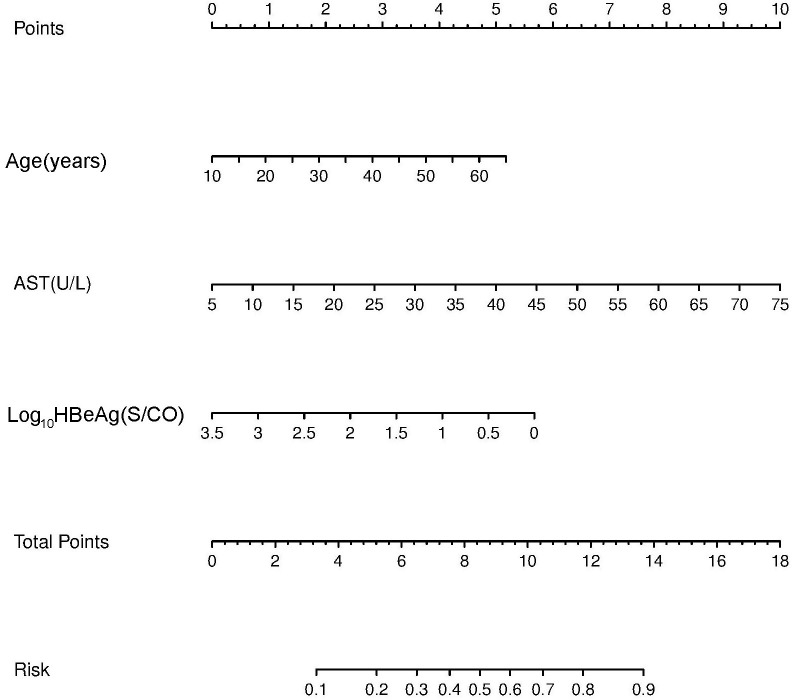
Non-minimal liver inflammation nomogram for hepatitis B e antigen (HBeAg)-positive chronic hepatitis B (CHB) patients with alanine aminotransferase (ALT) <80 U/L.

A nomogram was established based on the results obtained from the multivariate analysis. Each factor, including age, AST, and HBeAg, was assigned to a specific value in the corresponding scale axis and a line drawn upward to determine its score. The sum of the three scores resulted in a total score, and then a line was drawn downward to determine the probability of non-minimal liver inflammation in the risk axis for each patient (**Figure 1**).

### Validation of the prognostic nomogram for non-minimal liver inflammation

**Figure 2:**
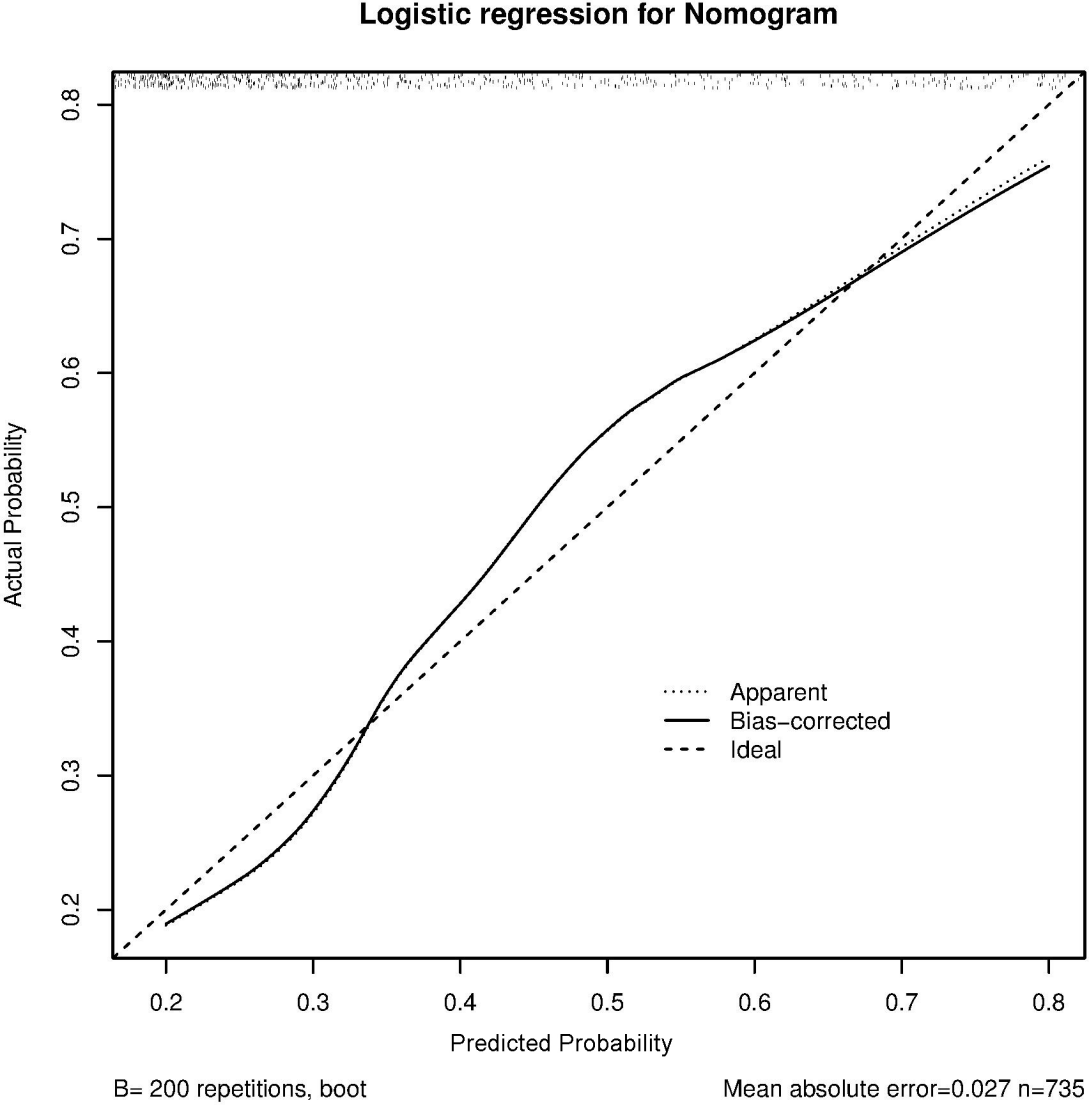
Calibration curve of non-minimal liver inflammation from the primary cohort.

**Figure 3:**
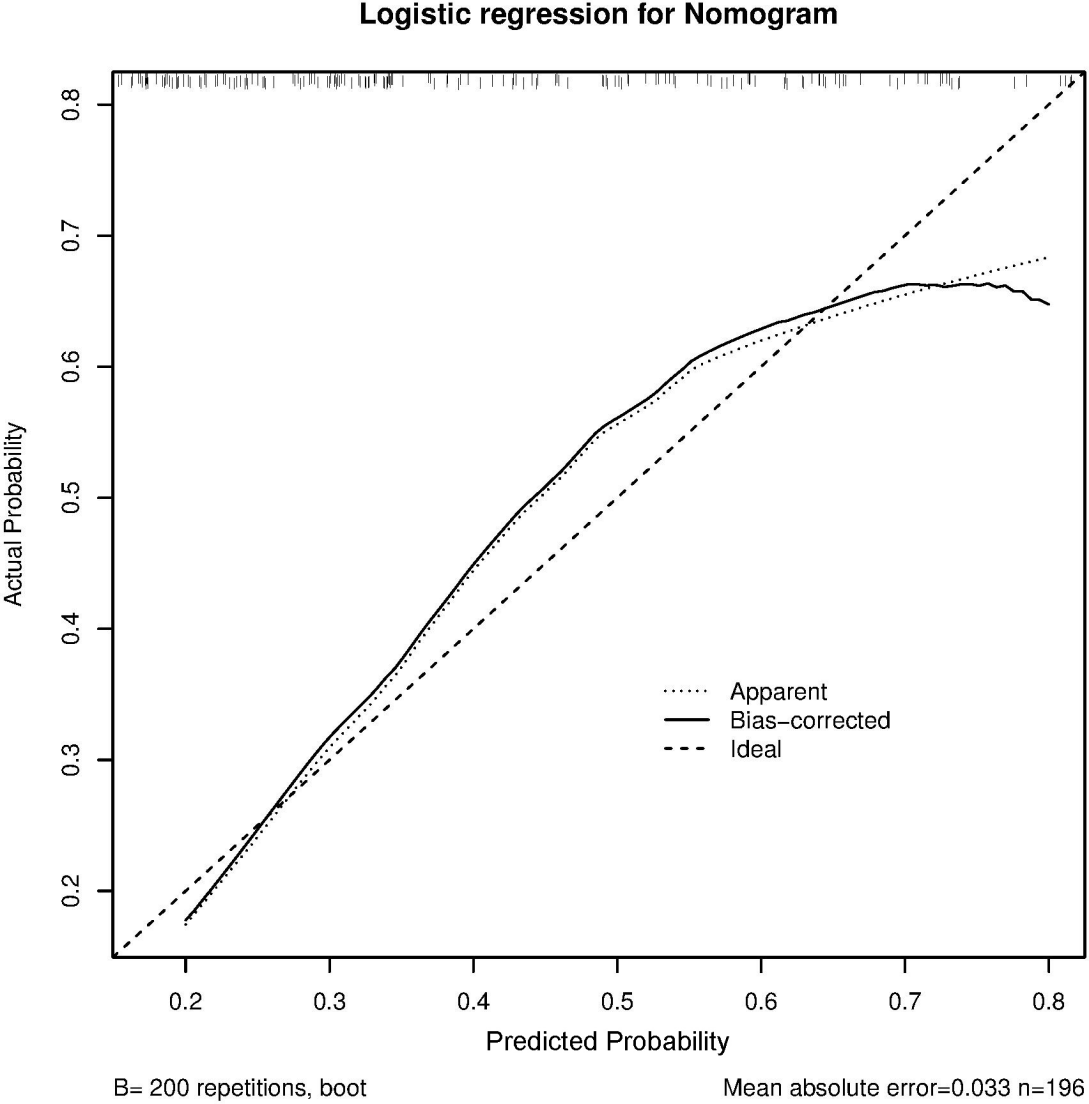
Calibration curve of non-minimal liver inflammation from the validation cohort.

The C-index and calibration curves were used to evaluate the predictive accuracy of the nomogram. The C-index of the nomogram from the primary cohort for predicting liver inflammation was 0.767 (95%CI = 0.734–0.802) (**Figure 2**), while that from the prospective validation cohort was 0.749 (95%CI = 0.681–0.817) (**Figure 3**). The calibration curves showed the best agreement between the predictive and observed probabilities in both the primary and prospective validation cohorts.

The predicted probability of the nomogram was plotted on the *x*-axis, while the actual probability was plotted on the *y*-axis. A dashed line along the 45°C line through the point of origin represented the perfect calibration model, where the predicted probabilities were identical to the actual probabilities.

## Discussion

Accurate assessment of liver inflammation in untreated patients with CHB infection has always been one of the focal points in HBV research. The purpose of this study was to establish a noninvasive liver inflammation diagnostic model mainly based on blood indicators.

In previous research studies, a decrease in the quantitative HBsAg and HBeAg to a certain cutoff value often indicates the onset of an immune clearance period (16, 19–21). Immune tolerance is gradually weakened with age (22, 23). The higher the ALT, the more obvious the liver inflammation. However, for patients with conflicting indicators, such as high ALT and HBsAg or low ALT and HBsAg, neither ALT nor HBsAg alone could make correct predictions (24, 25). Therefore, there is a need to develop a multifactorial scoring system for estimating liver inflammation that is simple and easy to use in untreated patients with CHB infection.

For patients with ALT > 2–5 ULN or above, liver inflammation was obvious or active, which marks the breakdown of immune tolerance. Deciding whether or not to start antiviral therapy in CHB patients with ALT < 2 ULN is a challenge for clinicians. There are pieces of evidence in favor of antiviral therapy. The results of liver biopsy can confirm that some of these patients have met the indication for antiviral therapy with obvious liver inflammation or liver fibrosis (21, 26–28). A subset of patients can progress from CHB directly to liver cancer even if ALT is normal (1–3). A timely antiviral treatment can reduce the HBV DNA load and decrease the incidence of liver cancer. Therefore, there is a need to establish a nomogram to identify patients with obvious liver inflammation and who need treatment from those HBeAg-positive CHB patients with ALT < 2 ULN.

The aim of this study was to identify non-minimal liver inflammation in treatment-naive CHB infection patients with ALT < 2 ULN with the help of a number of clinical noninvasive markers. Our results showed that AST, HBeAg, and age were independent variables for predicting noninvasive liver inflammation. The correlation between ALT or AST and liver inflammation has been well recognized by clinicians for a long time. Age affects the natural course of CHB infection, and it has been found that immune tolerance may be broken and that liver inflammation may occur when patients are over 30–40 years old. In our study, when age was over 30 and 40 years, non-minimal inflammation was found in 43.24% and 53.07% of patients with CHB infection and ALT < 2 ULN. There are many similar findings in the literature of quantitative HBeAg and HBsAg being both associated with HBV DNA or replicative covalently closed circular DNA (cccDNA) in HBeAg-positive patients (13, 21). In our research, we included AST instead of ALT into the prediction formula, considering that AST is a more sensitive factor for obvious liver inflammation of ALT < 2 ULN. Coincidentally, in the APRI (AST-to-PLT ratio index) formula for cirrhosis, AST was used, but not ALT. Similarly, HBeAg, but not HBsAg, was entered into the prediction formula. A possible explanation is that a decrease in HBeAg can better reflect the replication status of HBV or the breakdown of immune tolerance in untreated HBeAg-positive CHB patients.

To confirm the effect of the non-minimal liver inflammation model, we evaluated the predictive accuracy and discriminative ability of the nomogram using the C-index and calibration curves. In HBeAg-positive CHB patients, the calibration curve showed good agreement between the model predictions and the actual observations in both the primary and external validation cohorts. The C-index values were 0.767 (95%CI = 0.734–0.802) and 0.749 (95%CI = 0.681–0.817), respectively, indicating good predictive accuracy.

A nomogram is a visual prediction model. The predictors of liver inflammation in CHB patients with ALT < 2 ULN were shown in the nomogram. Nomograms were first used to evaluate the prognosis of cancer patients (29). They have been extended to other fields because of their reliability, visual intuition, and convenience (30, 31). A nomogram based on multivariate logistic regression analysis allows prediction of the diagnosis, stage, and prognosis of tumor or other diseases (32). Nomograms have been proven to be superior to risk stratification and artificial neural network models in predicting the prognosis of prostate cancer (33). The nomogram in this study revealed that liver inflammation becomes more obvious with age, corresponding to 2 at 30 years and 3 at 40 years. HBeAg of 1,000 S/CO represented 1, 100 S/CO represented 2, and 10 S/CO represented 4, indicating that the degree of liver inflammation increased with the decrease of quantitative HBeAg. When AST increased to 2 ULN, the effect on liver inflammation was more obvious. The probability of liver inflammation in CHB infection could be predicted by combining AST, age, and HBeAg. The total score was 10, corresponding to 0.7 in the nomogram, which means that the predicted probability of non-minimal liver inflammation was 70%. The effect of AST on the score was greater than that of age and HBeAg. AST > 2 ULN alone corresponded to a score of 10 and predicted the need for antiviral therapy. On the other hand, single indicators such as age and HBeAg could not exactly predict the need for antiviral treatment. The influence of the predictors age, HBeAg, and AST on liver inflammation was clearly reflected using this nomogram.

For patients with CHB infection, more effort has to be made on non-minimal liver inflammation model research. At present, there are many non-invasive prediction models for chronic hepatitis, most of which are primarily for predicting liver fibrosis, such as APRI and FIB-4 (Fibrosis-4) (34), with a few models for predicting liver inflammation (24). Liver inflammation usually occurs earlier than liver fibrosis; therefore, early diagnosis of liver inflammation in CHB infection is more important and is difficult. Chang et al. and other researchers have reported a prediction model of CHB infection whose scope is the histological progression of liver diseases, i.e., significant liver fibrosis and/or liver inflammation (25, 28, 35). The model developed in this study is a noninvasive prediction model for the identification of non-minimal liver inflammation in patients with CHB infection. This study did not involve fibrosis evaluation and is therefore not fit for comparison with APRI or FIB-4. In untreated HBeAg-positive CHB patients, the liver inflammation caused by CHB is related to the balance between the immune system and HBV infection. Quantitative changes in several indicators, such as HBsAg and HBeAg, can signal the breakdown of immune tolerance and the start of immune clearance in CHB infection. It is necessary to include immune-related indicators in the inflammatory prediction model for CHB infection. In general, ALT or AST is included in most models of liver inflammation or fibrosis. It has been found that the predictive ability of AST is better than that of ALT. HBV DNA, HBsAg, and HBeAg can reflect the replication ability of HBV in untreated CHB patients. HBsAg and HBeAg have been previously found to be negatively correlated with liver inflammation. In this study, HBsAg, HBeAg, and HBV DNA were all included, and HBeAg was found to have better predictive ability. Compared with other models, the significance of this non-minimal liver inflammation model is that it confirmed the importance of HBeAg in the identification of inflammation. On the one hand, HBeAg can reflect the replication level of HBV in HBeAg-positive CHB patients; on the other hand, a decline in HBeAg is often an early sign of the breakdown of immune tolerance (36).

The advantages of the non-minimal liver inflammation nomogram developed in this study are as follows: 1) it can be calculated and predicted repeatedly at different time points of the disease, which is convenient for long-term prospective assessment of liver inflammation in HBeAg-positive CHB patients; 2) the alignment chart of the nomogram is visual and practical, which is beneficial for doctors to make decisions on when to start antiviral treatment for individual patients, and it is also suitable for multifactor comprehensive prediction of liver inflammation; 3) this nomogram has been verified in the prospective validation cohort; and 4) the nomogram included HBeAg, a replication- and an immune-related marker of HBV. This study has several limitations. Firstly, the data were from Chinese CHB patients, whose genotypes are mostly of B and C types. Secondly, the sample size was relatively small, and the nomogram was unable to predict liver inflammation in patients with CHB infection according to gender.

In conclusion, the non-minimal liver inflammation nomogram that included age, AST, and HBeAg index has been well established and verified in HBeAg-positive CHB patients with ALT < 2 ULN. The nomogram could help in predicting the probability of non-minimal liver inflammation. The predictive accuracy of this nomogram was confirmed using the C-index and calibration curves.

## Data availability statement

The raw data supporting the conclusions of this article will be made available by the authors, without undue reservation.

## Ethics statement

The studies involving human participants were reviewed and approved by the Ethics Committee of Beijing Ditan Hospital. The patients/participants provided written informed consent to participate in this study.

## Author contributions

ML and YX contributed to the study concept and design. XB, QX, LuZ, XC, and WC conducted the experiments and collected the data. YanL, GS, TJ, WD, and SW collected patient information. ShiW, LuxZ, LY, ShuW, MX, and YG ordered the reagents and materials. YaoL performed the statistical analyses and wrote the first draft. HH, SW, RL, MC, MX, LH, and XC edited the English version. All authors contributed to the article and approved the submitted version.
